# Are *pre-miR-146a* and *PTTG1* associated with papillary thyroid cancer?

**DOI:** 10.1530/EC-13-0066

**Published:** 2013-11-18

**Authors:** Marco Marino, Valentina Cirello, Valentina Gnarini, Carla Colombo, Elisa Pignatti, Livio Casarini, Chiara Diazzi, Vincenzo Rochira, Katia Cioni, Bruno Madeo, Cesare Carani, Manuela Simoni, Laura Fugazzola

**Affiliations:** 1 Unit and Chair of Endocrinology and Metabolism, NOCSAE, Department of Biomedical Metabolic and Neural SciencesUniversity of Modena and Reggio EmiliaVia Pietro Giardini 135541126, ModenaItaly; 2 Center for Genomic ResearchUniversity of Modena and Reggio EmiliaModenaItaly; 3 Department of Clinical Sciences and Community HealthUniversity of MilanMilanItaly; 4 Endocrine UnitFondazione IRCCS Ca' Granda MilanMilanItaly; 5 Azienda USL of ModenaModenaItaly

**Keywords:** *PTTG1*, miRNA, *pre-mir-146a*, polymorphism, SNP, papillary thyroid carcinoma, rs2910164, rs1862391, rs2910202

## Abstract

Papillary thyroid carcinoma (PTC) is the most common endocrine malignancy, with a steadily increasing incidence in the last few decades worldwide. The predisposition to developing this carcinoma by the heterozygous state of rs2910164 within the precursor of the *miR-146a* has been reported, but recently not confirmed. Interestingly, on the same chromosome, almost 50 kb separate the *pre-miR-146a* from the pituitary tumor-transforming gene 1 (*PTTG1*), a proto-oncogene involved in several tumors, including thyroid cancers. In this study, we analyzed, using a case–control design, the genetic association between PTC and the genomic region encompassing *pre-miR-146a* rs2910164 and *PTTG1* rs1862391 and rs2910202. We enrolled 307 affected patients and 206 healthy controls. The possible presence of thyroid nodules in controls was excluded by ultrasonography. All the cases were submitted to single-nucleotide polymorphism (SNP) genotyping of *pre-miR-146a* and *PTTG1*, and risk association analyses were carried out. The genotypic and allelic frequencies of *pre-miR-146a* rs2910164 were not statistically different in the patients and controls, and this SNP was not in linkage disequilibrium with the investigated *PTTG1* SNPs. Consistently, meta-analyses, the first including all the affected cases published to date, did not confirm the previously reported association of the heterozygous CG genotype with PTC. The *PTTG1* SNPs exhibited the same allelic frequency in the patients and controls and were not associated with the disease. In conclusion, in a well-selected Italian population, neither *pre-miR-146a* rs2910164 nor *PTTG1* rs1862391 and rs2910202 were found to be associated with the risk of developing PTC.

## Introduction

Thyroid cancers can be well-differentiated, as papillary and follicular subtypes, or poorly differentiated and anaplastic carcinomas. Papillary thyroid carcinoma (PTC) represents up to 80% of all the thyroid cancer cases. Various molecular alterations have been reported to be associated with PTC, including *BRAF* (40–45%) and *RAS* (10–20%) point mutations and *RET* (10–20%) or *TRK* (*NTRK1*) (<5%) rearrangements, all involved in the activation of the MAPK pathway (*NTRK1*) (1, 2). Although the literature has a large amount of information on these alterations, there are a few cases of PTC that are not associated with the mutations mentioned above. To fill this gap, the scientific community is still actively searching for new diagnostic and prognostic molecular markers of PTC. In the last 8 years, studies on PTC have been focused on microRNAs (miRNAs), a class of endogenous, small noncoding RNA molecules that function as negative regulators of the expression of protein-encoding genes, involved in many cellular processes such as development, apoptosis, and proliferation (3, 4). The first evidence for a potential role for several miRNAs in PTC was the different expression of some of these miRNAs in tumor tissue, compared with unaffected tissue of the thyroid gland (4). In particular, two studies have shown the association between *pre-miR-146a* rs2910164 and PTC, evaluating the impact of this single-nucleotide polymorphism (SNP) on the regulation of target mRNAs (5, 6). As a conclusion, these studies reported an association between the *pre-miR-146a* CG genomic variant (rs2910164) and the predisposition to developing PTC. However, further case–control studies, including Caucasian and Asian populations, have been unable to confirm this association (7, 8). Therefore, the involvement of the genomic variant *pre-miR-146a* rs2910164 in thyroid cancers remains unclear.

Interestingly, on the same chromosome 5, ∼50 kb separate *pre-miR-146a* (OMIM*610566) from the pituitary tumor-transforming gene 1 (*PTTG1*) (OMIM*604147), reported to be overexpressed in thyroid carcinomas (9, 10, 11). *PTTG1*, encoding a securin homolog, is involved in the physiological control of mitosis, DNA repair, gene regulation, and fetal development (12, 13). When experimentally mutated, this gene has been shown to promote the proliferation of cancer, to repress the reparation of double-stranded DNA breaks, and to interact with different factors, possibly driving the progression of the disease (13). It has recently been shown that the genomic region on chromosome 5, encompassing *pre-miR-146a* and *PTTG1*, is associated with systemic lupus erythematosus (SLE) (12, 14) and cancers (15, 16, 17), including papillary thyroid tumor (5, 6, 7, 8, 10). The aim of the present study was to analyze this region and its association with PTC in a well-selected case–control population of the Northern area of Italy, adopting, as genetic markers, the rs2910164 SNP of *pre-miR-146a* and, for the first time, the rs1862391 and rs2910201 SNPs of *PTTG1*. The latter gene was selected both for its involvement in cancers and for its genomic proximity to *pre-miR-146a*, making it a possible ‘true’ candidate for the genomic association with PTC previously inconsistently found with *pre-miR-146a*.

In addition, we carried out meta-analyses on the data reported in the literature (6, 7, 8), including our data, considering Caucasian and Asian populations.

## Subjects and methods

### Subjects

Using a case–control study design, 307 patients (83 males and 224 females) diagnosed as having PTC and 206 healthy control subjects (75 males and 131 females), all of Caucasian origin and Italian ethnicity, were enrolled. The majority of cases (192/307) were recruited at the Endocrine Unit of the University of Modena and Reggio Emilia, while 115 patients were recruited at the Endocrine Unit of the University of Milan. Tumors were classified according to the thyroid malignancy World Health Organization classification and staged according to the sixth edition of TNM staging (18). Controls, 137/206 from Modena and 69/206 from Milan, were selected among the volunteers. Importantly, to be enrolled, controls had to have a normal thyroid function and a normal ultrasound scan, excluding autoimmune or nodular diseases. The total number of 307 patients and 206 controls satisfied the criteria of the power analysis, requiring a minimum sample size of 164 patients. Written informed consent was obtained from all the participants, who donated a blood sample for association studies on thyroid cancers. The study protocol was approved by the Local Ethics Committee (file no. 122/08).

### Criteria for the selection of SNPs

We selected the rs2910164 SNP of the *pre-miR-146a* gene because of its direct association with PTC, as reported in the literature (5, 6, 7, 8).

**Figure 1:**
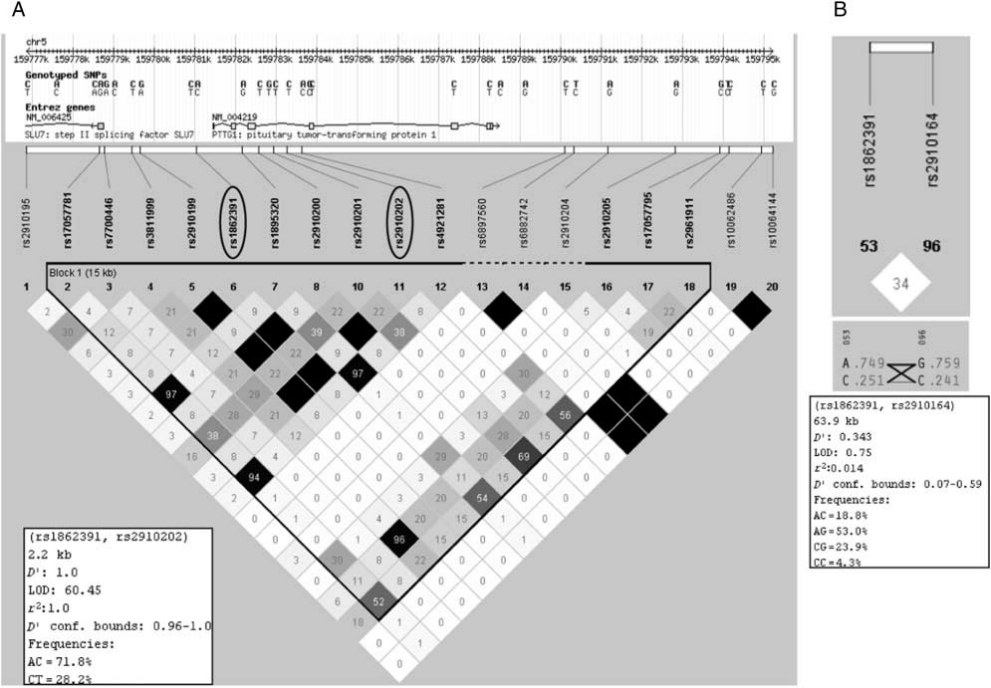
(A) Haploview window showing the *PTTG1* gene and its LD block, containing the two selected SNPs, rs1862391 and rs2910202. The percentage of LD is shown in each diamond. Black diamonds without a number correspond to 100% of LD (*D*′=1.0, *r*^2^=1). (B) Results of the analysis of LD between *PTTG1* rs1862391 and *pre-miR-146a* rs2910164 showing a distance of 63.9 kb between the two SNPs and a very low percentage of LD, 34% (*D*′=0.343, *r*^2^=0.014).

For *PTTG1*, four different SNPs have been considered in previous studies. Brendle *et al*. (19) investigated rs1862391, rs1862392, and rs2961952 in breast cancer, whereas Löfgren *et al*. (12) and Yang *et al*. (20) described rs2431697 associated with SLE and psoriasis. Based on this information, we decided to analyze the genomic region corresponding to *PTTG1*, including these four SNPs, but using other, more informative genetic markers following the guidelines of Pettersson *et al*. (21) for genetic association studies. The online database ‘HapMap’ (http://hapmap.ncbi.nlm.nih.gov) contains 32 annotated SNPs in the region of over 10 kb encompassing the whole *PTTG1* gene. These SNPs were analyzed using Haploview 4.2 (Harvard University and Massachusetts Institute of Technology, USA) to verify those falling within a block of linkage disequilibrium (LD), using the LD parameters *D*′ and *r*^2^ (21). By considering the minor allele frequency (MAF) available in HapMap and the degree of allelic correlation between LD blocks, two SNPs were identified as the best haplotype marker candidates (*D*′=1 and *r*^2^=1; Fig. 1A): rs1862391 (A/C) and rs2910202 (C/T). These SNPs exhibit the same MAF, corresponding to the highest MAF among all the SNPs belonging to the *PTTG1* LD block (29% for Utah residents with Northern and Western European ancestry from the CEPH collection, CEU; Fig. 1A). Of these, rs1862391 had been studied previously in breast cancer cases (19). Our analysis revealed several criteria of inadequacy for the other three SNPs studied previously in the literature, such as low probability of LD and different MAF (between rs1862391 and rs1862392) and the exclusion from LD blocks (rs2961952 and rs2431697). Particularly, the rs2431697 SNP neither belongs to *PTTG1* nor falls within the *PTTG1* LD block; furthermore, the distance between this SNP and the gene is over 23 kb. The rs1862391 SNP is located at about 500 bp upstream the first exon, inside the promoter region, while the rs2910202 SNP lies about 400 bp downstream exon 3 (between exons 3 and 4). A distance of 2.2 kb separates the two selected *PTTG1* SNPs (Fig. 1A), while a distance of 63.9 kb separates the rs1862391 SNP of *PTTG1* from the rs2910164 SNP of *pre-miR-146a* (Fig. 1B).

### Genomic DNA analysis

DNA was extracted from the whole peripheral blood of all the patients and controls, using the Nucleon BACC2 Kit (GE Healthcare, Little Chalfont Buckinghamshire, UK). To genotype the *pre-miR-146a* rs2910164 SNP, at least 100 ng of DNA were PCR-amplified using specific intronic primers in a One-Advanced Thermocycler (Euroclone S.p.A., Life Sciences Division, Siziano, Italy), according to the following protocol: 98 °C for 5 min followed by 35 cycles of 98 °C for 1 min, 58 °C for 1 min, and 72 °C for 2 min with a final step of 72 °C for 10 min. The PCR products were directly sequenced after the removal of the unincorporated dNTPs and primers using ExoSAP-IT (USB Products Affymetrix, Inc., Cleveland, OH, USA). An aliquot of 3–10 ng/100 bp of purified DNA and 3.2 pmol of either the forward or reverse primer was used in standard cycle sequencing reactions with ABI PRISM BigDye Terminators and run on the ABI PRISM 310 Genetic Analyzer (PE Applied Biosystems). The cycle sequencing conditions consisted of 25 cycles of 96 °C for 30 s, 50 °C for 15 s, and 60 °C for 4 min. One sequence read from each direction across the entire coding region and including intron–exon boundaries was obtained for each sample. All the primers used for PCR and sequences are listed in Supplementary Table 1, see section on supplementary data given at the end of this article. Electropherograms were analyzed using the Chromas Software (http://technelysium.com.au).

Genotyping for the two *PTTG1* SNPs, rs1862391 and rs2910202, was carried out using the High Resolution Melting (HRM) technology on a CFX96 Real-time PCR detection system (Bio-Rad Laboratories). The Beacon Designer 7.91 Software (Bio-Rad) was used to design and test, *in silico*, all the HRM primers (Supplementary Table 1). To conduct HRM assays, 20 ng of genomic DNA were amplified in a final volume of 15 μl, containing 1× SsoFast Eva Green Supermix (Bio-Rad) and 0.5 μmol/l of each primer. The conditions of qPCR were as follows: 98 °C for 2 min followed by 39 cycles of 3 s at 98 °C and 5 s at 58.2 °C (for rs1862391) or 58.2 °C (for rs2910202). After the amplification step, the PCR products were denatured at 98 °C for 30 s and slowly renatured at 65 °C for 90 s. A high-resolution melt was immediately carried out with a progressive denaturation step from 67 to 80 °C (for rs1862391) or from 66 to 84 °C (for rs2910202), by increasing the temperature by 0.2 °C every 10 s. All the HRM raw data (preliminary melting curves) were analyzed using the specific software CFX Manager (Bio-Rad) and Precision Melt Analysis (Bio-Rad). Uncertain results were verified through Sanger sequencing using the 4-capillary ABI Prism 3130 Genetic Analyzer (Applied Biosystems). The FastPCR 6.0 Software (Primer Digital Ltd., Helsinki, Finland) (22) was used to design the PCR and sequence primers for the two *PTTG1* SNPs (Supplementary Table 1). Electropherograms were analyzed using the Sequencing Analysis 5.3.1 Software (Applied Biosystems). The accuracy of *pre-miR-146a* rs2910164 genotyping was assessed by repeating the genotyping analysis for 40 randomly chosen samples. The accuracy of *PTTG1* SNP genotyping was assessed by the perfect LD between the two SNPs, as expected in CEU. The success rate of genotyping was 100%.

### Statistical analyses

To assess the minimum sample size for the present study to be valid, a power analysis was carried out using the GPower 3.1.6 Software (Dusseldorf, Germany) (effect size *w*=0.30; *α*=0.05; power=0.97), following the programmer's instructions (23). The *χ*^2^ test was used to assess the deviation from the Hardy–Weinberg equilibrium (HWE) in the control group and to analyze the differences in genotype distribution and allelic frequencies between the patients and controls. The odds ratio (OR) and its 95% CI were used to assess the strength of the association between the analyzed polymorphisms and the disease. In meta-analyses, heterogeneity between all the analyses was determined by *I*^2^, and data with high heterogeneity (*I*^2^ >50%) were processed using a random-effects model or a fixed-effects model (24). All the statistical analyses were carried out using the GraphPad Prism 5 Software (www.graphpad.com), and the Review Manager 5.2 Software (Copenhagen, Denmark) was used for the meta-analyses. *P* value <0.05 was considered to be statistically significant.

## Results

The genotype results did not deviate from the HWE compared with those of the HapMap CEU population (*P*=0.3517 for *pre-miR-146a* and *P*=0.4084 for *PTTG1*). In addition, the statistical analyses revealed no differences in genotype distribution between the patients enrolled in Modena and those enrolled in Milan (*P*=0.3650 for *pre-miR-146a* and *P*=0.3684 for *PTTG1*). Similarly, no differences were evident between controls recruited at the two sites (*P*=0.9193 for *pre-miR-146a* and *P*=0.8492 for *PTTG1*); thus, all the data of patients and controls were assessed together.

No significant differences in genotype distribution and allelic frequencies of *pre-miR-146a* rs2910164 were found between the patients and controls (Table 1). The OR analysis did not reveal any significant risk association between the CC or GG genotype and PTC (Table 1). The previously described association of the CG genotype with PTC (5, 6) was not confirmed (Table 1).

**Figure 2:**
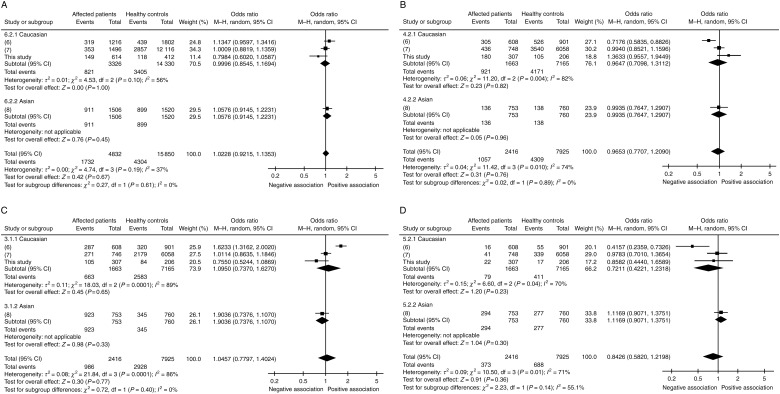
Results of the meta-analyses for the association risk between *pre-miR-146a* rs2910164 and PTC. All the meta-analyses included the Caucasian and Asian ethnic subgroups. All the considered studies and all the results of the meta-analyses are represented by the forest plot. The size of the squares indicates the weight of each individual study. The diamond on the top corresponds to the result of the meta-analyses, applied to the Caucasian subgroup, Asian subgroup, or both subgroups. M–H, Mantel–Haenszel method. (A) The plot, showing an OR of 1.0228, presents the association risk between the alleles (C vs G) of *pre-miR-146a* rs2910164 and papillary cancer and indicates an association that is not significant. (B) The plot, showing an OR of 0.9653, presents the association risk between the GG genotype of *pre-miR-146a* rs2910164 and papillary cancer and indicates an association that is not significant. (C) The plot, showing an OR of 1.0457, presents the association risk between the CG genotype of *pre-miR-146a* rs2910164 and papillary cancer and indicates an association that is not significant. (D) The plot, showing an OR of 0.8426, presents the association risk between the CC genotype of *pre-miR-146a* rs2910164 and papillary cancer and indicates an association that is not significant.

All the meta-analyses were carried out by combining the results of the present study with the data of independent association studies from the literature (6, 7, 8). The meta-analyses did not detect an association either between *pre-miR-146a* rs2910164 alleles and PTC (Fig. 2A) or between genotypes and PTC (Fig. 2B, C and D).

For *PTTG1*, the perfect LD between the rs1862391 and rs2910202 SNPs was confirmed. The genotypic and allelic frequencies of the patients and controls were not different (Table 2). No risk associations were found between the two *PTTG1* SNPs and PTC (Table 2). The degree of LD between *pre-miR-146a* rs2910164 and the chosen *PTTG1* SNPs was low (34%, Fig. 1B).

Since individual genotypes, belonging to two or more SNPs, often were not informative, we extended the analysis by assessing the risk associated with the nine possible diplotypes, arising from the combination of all genotypes of the two SNPs, *pre-miR-146a* rs2910164 and *PTTG1* rs1862391 (Table 3). This analysis did not reveal any specific diplotype to be associated with PTC (Table 3).

## Discussion

In recent years, scientific research has been increasingly orientated to understanding the physiological role and implication of miRNAs in several cancers. Conflicting results exist about the role of *pre-miR-146a* rs2910164 in thyroid cancers. Indeed, although a significant association between the heterozygous CG genotype of rs2910164 and PTC has been reported, investigating three different Caucasian populations (from Finland, Poland, and USA) (6), these data have not been confirmed in two series of Caucasian (7) and Asian (8) origins. In particular, by comparing patients with benign thyroid tumors with patients with PTC, Wei *et al*. (8) reported that the GG genotype rather than the CG genotype represented a significant risk factor associated with malignant transformation in a Chinese Han population. In the present study, neither the *pre-miR-146a* rs2910164 SNP nor the *PTTG1* rs1862391 and rs2910201 SNPs were found to be associated with PTC. The differences between the results of the present study and those of the study carried out by Jazdzewski *et al*. (6) could be due to the different number of patients and controls enrolled, which was lower in the present study. Nevertheless, discordant results have also been obtained in the three larger series published by Jazdzewski *et al*. (6), Jones *et al*. (7), and Wei *et al*. (8), including 608, 748, and 753 PTC cases respectively. Another possibility could lie in the selection of controls, which has previously been carried out based on only anamnestic criteria. By contrast, in the present study, the control subjects were screened and selected on the basis of a normal thyroid ultrasound scan. Owing to the high prevalence of nodular diseases worldwide, and in particular in regions such as Italy, with a mild iodine deficiency, several subjects were excluded due to the incidental demonstration of nodules of any dimension in the absence of clinical signs and symptoms. In particular, we have recently shown that thyroid abnormalities such as nodular goiter and thyroiditis are present in 50.3% of asymptomatic subjects from in Italy (25). Moreover, differentiated thyroid cancer was found in 1% of the screened, asymptomatic subjects and in 2% of those affected by nodular goiter (25). Since previous association studies did not select the control group on the basis of a normal thyroid ultrasound scan (6, 7, 8), it is tempting to speculate that differences in the selection criteria might explain at least in part the discrepancies of the literature.

In such cases, meta-analyses may be helpful. In several published studies, meta-analyses have been carried out to clarify the actual association among polymorphic variants and cancers or other diseases (24, 26, 27). Recently, this kind of analyses has been carried out for evaluating the association between the *pre-miR-146a* rs2910164 SNP and over 15 different types of cancers (26, 28, 29, 30, 31, 32, 33). In the present study, meta-analyses focused only on the possible association between *pre-miR-146a* rs2910164 and PTC have been carried out for the first time. Caucasian and Asian ethnic subgroups were considered, separately or together, and the risk association according to the different alleles and genotypes (Fig. 2A, B, C and D) was analyzed. Our meta-analyses included all the studies based on rs2910164 in PTC carried out so far. To the best of our knowledge, no genome-wide association studies on PTC have focused on rs2910164, rs1862391, or rs2910202 so far. Our meta-analyses, including a total of 2416 patients and 7925 controls, did not confirm the association of *pre-miR-146a* rs2910164 with PTC using any of the models analyzed (dominant or recessive). Therefore, we must conclude that, according to the current knowledge, the genotypes of *pre-miR-146a*, as assessed in peripheral blood leukocytes, are not associated with PTC (Fig. 2B, C and D).

Given the proximity of *PTTG1* to *pre-miR-146a* and its postulated role in cancers, we extended our analyses to this gene. We reasoned that *PTTG1* could be associated with PTC, since a previous study had reported the association of the rs2431697 SNP, located in the intergenic region dividing the *PTTG1* and *pre-miR-146a* genes, with SLE (12). Moreover, several studies have shown the involvement of *PTTG1* in the onset and/or progression of different types of tumors (10, 11, 13, 15, 16, 17), including thyroid cancers (10). The postulated role of securin, the protein encoded by *PTTG1*, in thyroid tumorigenesis and in the development of hyperplastic lesions could be due to its overexpression during metaphase–anaphase transition (10), which could generate aneuploidy (10, 13). Taking all these data into consideration, we decided to explore the association of *PTTG1* with PTC, using, for the first time, the rs1862391 A/C and rs2910201 C/T SNPs as genetic markers. The *PTTG1* SNPs (rs1862391 and rs2910202), in perfect LD, exhibited the same allelic frequency in patients and controls and were not associated with PTC, probably excluding a possible role of this gene in thyroid tumorigenesis.

In conclusion, the present case–control study, in a well-selected population of the Northern area of Italy, and the meta-analyses, combining the results of all the association studies carried out so far, demonstrate that *pre-miR-146a* rs2910164 is not associated with PTC. In particular, no genotype was found to be associated with an increased risk of developing PTC, including the previously suggested heterozygous CG genotype. Extending the genetic analysis to the contiguous genomic region, we showed that *PTTG1* was not associated with PTC. In the light of these data, the genomic region encompassing *pre-miR-146a* and *PTTG1* is probably not associated with the predisposition to developing PTC. The occurrence of *PTTG1* gene mutations in PTC patients and the role of somatic mutations of both the investigated genes remain to be explored.

## Supplementary data

This is linked to the online version of the paper at http://dx.doi.org/10.1530/EC-13-0066.

## Author contribution statement

M Marino and V Cirello contributed equally to this work.

## Figures and Tables

**Table 1:** Genotype distribution and allelic frequencies of *pre-miR-146a* rs2910164 in PTC cases and controls.

**rs2910164**	**PTC cases** (*n* (%))	**Controls** (*n* (%))	***χ*^2^**	**Odds ratio** (%)	**95% CI**	***P* value**
Total *n*	307	206				
Genotype			*P*=0.2299			
GG	180 (58.6)	105 (51)		1.363 (GG vs CG+CC)	0.9556–1.945	0.0869
CG	105 (34.2)	84 (40.8)		0.755 (CG vs GG+CC)	0.5243–1.087	0.1302
CC	22 (7.2)	17 (8.2)		0.8582 (CC vs GG+CG)	0.4439–1.659	0.649
Allele			*P*=0.1175			
C	149 (24.3)	118 (28.6)		Reference		
G	465 (75.7)	294 (71.4)		1.253 (G vs C)	0.9445–1.661	0.1175

**Table 2:** Genotype distribution and allelic frequencies of *PTTG1* rs1862391 in PTC cases and controls.

**rs1862391**	**PTC cases** (*n* (%))	**Controls** (*n* (%))	***χ*^2^**	**Odds ratio**	**95% CI**	***P* value**
Total *n*	307	206				
Genotype			*P*=0.9798			
AA	185 (60.7)	123 (59.7)		1.023 (AA vs AC+CC)	0.7137–1.467	0.9005
AC	101 (32.6)	68 (33)		0.995 (AC vs AA+CC)	0.6834–1.449	0.9791
CC	21 (6.7)	15 (7.3)		0.935 (CC vs AA+AC)	0.4701–1.860	0.8479
Allele			*P*=0.8541			
C	143 (23.3)	98 (23.8)		Reference		
A	471 (76.7)	314 (76.2)		1.028 (A vs C)	0.7660–1.379	0.8541

**Table 3:** Odds ratios (ORs) for the risk of developing PTC based on genotype combinations of rs2910164 (*pre-miR-146a*) and rs1862391 (*PTTG1*).

**Diplotype**	**rs2910164**	**rs1862391**	**PTC cases** (*n* (%))	**Controls** (*n* (%))	**OR**	**95% CI**	***P* value**
Total *n*			307	206			
1	GG	AA	101 (32.9)	56 (27.2)	1.313 (diplotype 1 vs all)	0.8905–1.937	0.2009
2	GG	AC	65 (21.2)	40 (19.4)	1.115 (diplotype 2 vs all)	0.7174–1.732	0.6566
3	GG	CC	14 (4.6)	9 (4.4)	1.046 (diplotype 3 vs all)	0.4440–2.464	1.0000
4	CG	AA	68 (22.1)	55 (26.7)	0.7811 (diplotype 4 vs all)	0.5185–1.177	0.2472
5	CG	AC	31 (10.1)	24 (11.7)	0.8518 (diplotype 5 vs all)	0.4841–1.499	0.6806
6	CG	CC	6 (2)	5 (2.4)	0.8013 (diplotype 6 vs all)	0.2412–2.662	0.9589
7	CC	AA	16 (5.2)	12 (5.8)	0.8889 (diplotype 7 vs all)	0.4114–1.921	0.9190
8	CC	AC	5 (1.6)	4 (1.9)	0.8361 (diplotype 8 vs all)	0.2218–3.152	0.9376
9	CC	CC	1 (0.3)	1 (0.5)	0.6699 (diplotype 9 vs all)	0.04164–10.78	0.6613
